# Parasitic nematodes simultaneously suppress and benefit from coccidian coinfection in their natural mouse host

**DOI:** 10.1017/S0031182019000192

**Published:** 2019-03-27

**Authors:** Melanie Clerc, Andy Fenton, Simon A. Babayan, Amy B. Pedersen

**Affiliations:** 1 Institute of Evolutionary Biology and Centre for Immunity, Infection and Evolution, School of Biological Sciences, University of Edinburgh, Edinburgh UK, EH9 3FL, UK; 2 MRC Centre for Inflammation Research, The Queenʼs Medical Research Institute, University of Edinburgh UK, EH16 4TJ, UK; 3 Institute of Integrative Biology, University of Liverpool, Liverpool UK, L69 7ZB, UK; 4 Institute of Biodiversity, Animal Health & Comparative Medicine, University of Glasgow, Glasgow UK, G12 8QQ, UK

**Keywords:** Coinfection, *Eimeria hungaryensis*, *Heligmosomoides polygyrus*, laboratory systems, wild mice

## Abstract

Within-host interactions among coinfecting parasites are common and have important consequences for host health and disease dynamics. However, these within-host interactions have traditionally been studied in laboratory mouse models, which often exclude important variation and use unnatural host–parasite combinations. Conversely, the few wild studies of within-host interactions often lack knowledge of parasite exposure and infection history. Here we exposed laboratory-reared wood mice (*Apodemus sylvaticus*) that were derived from wild-caught animals to two naturally-occurring parasites (nematode: *Heligmosomoides polygyrus*, coccidia: *Eimeria hungaryensis*) to investigate the impact of coinfection on parasite infection dynamics, and to determine if the host immune response mediates this interaction. Coinfection led to delayed worm expulsion and prolonged egg shedding in *H. polygyrus* infections and lower peak *E. hungaryensis* oocyst burdens. By comparing antibody levels between wild and colony-housed mice, we also found that wild mice had elevated *H. polygyrus*-IgG1 titres even if currently uninfected with *H. polygyrus*. Using this unique wild-laboratory system, we demonstrate, for the first time, clear evidence for a reciprocal interaction between these intestinal parasites, and that there is a great discrepancy between antibody levels measured in the wild *vs* those measured under controlled laboratory conditions in relation to parasite infection and coinfection.

## Introduction

Individuals in their natural environment can be exposed to and infected with a multitude of parasites, either sequentially or simultaneously (Pedersen and Fenton, 2007; Telfer *et al*., 2008), which can lead to within-host interactions between parasite species (Graham, 2008; Seabloom *et al*., 2015). While interactions between microparasites (bacteria, protozoa, viruses) and macroparasites (e.g. helminths) can positively and negatively impact the outcome of disease (Salgame *et al*., 2013), the direction of within-host interactions due to coinfection can be variable, resulting in both positive (facilitating) and negative (competitive) interactions. In addition, host demographic (e.g. sex, age, reproductive condition) and environmental variation can affect the strength and direction of parasite interactions (Moreno *et al*., 2013; Gorsich *et al*., 2014). Therefore, by developing an understanding of what determines the magnitude and direction of parasite interactions can help to alleviate host damage and guide new trajectories for improved treatment and control programmes (Pedersen and Fenton, 2007; Maizels *et al*., 2012).

Most studies which have investigated both the mechanisms and health impacts of within-host parasite interactions have used highly controlled laboratory environments, mainly utilising inbred laboratory mice raised under standard pathogen-free conditions (Graham, 2008; Knowles, 2011). However, several recent studies have highlighted the importance of making the laboratory mouse model better reflect natural systems by introducing wild mouse microbiomes (Rosshart *et al*., 2017), infection and coinfection (Maizels and Gause, 2014; Reese *et al*., 2016) and by co-housing mice with pet-shop mice to allow exposure to pathogens and microbes (Beura *et al*., 2016). For example, Beura *et al*. (2016) have shown that the immune cell composition of feral and pet-shop mice resembles that of human adults, whereas laboratory mice show very low levels of differentiated memory T-cells, similar to human neonates (Beura *et al*., 2016). As a result of the often unrealistic conditions used in laboratory studies, and the accompanying difficulty in translating these results to the real world, researchers have begun to investigate the underlying mechanisms of parasite within-host interactions in natural environments. These studies have helped unravel the natural conditions under which parasites interact and importantly what factors determine the impacts of these interactions on host health and disease (Ezenwa *et al*., 2010; Ezenwa and Jolles, 2011; Turner *et al*., 2011; Friberg *et al*., 2013; Knowles *et al*., 2013; Pedersen and Antonovics, 2013). However, these studies are also not without limitations, as both the exposure and infection history of wild animals is not usually known, which leads to uncertainty in understanding the causal relationships between parasite infection/exposure and host characteristics such as an animal's immune phenotype (Abolins *et al*., 2011). Further, wild animals often experience resource limitation at various times throughout their lives, which can potentially reduce their investment in specific aspects of their life-history, for example, in the development of their immune system (Tate and Graham, 2015). To overcome the limitations of both laboratory and wild studies, and to better understand the causes and consequences of interactions between coinfecting parasites, field and laboratory approaches need to be better integrated.

Our previous field experiments revealed a negative interaction between two gastrointestinal parasites of wild wood mice (*Apodemus sylvaticus*), the nematode *Heligmosomoides polygyrus* and the coccidian microparasite *Eimeria hungaryensis*. We found that a single dose of the anthelmintic drug Ivermectin reduced *H. polygyrus* prevalence by 70%, but consequently led to a 15-fold increase in the burden of coinfecting *E. hungaryensis* 1–3 weeks post-treatment (Knowles *et al*., 2013). However, it is currently unknown whether this interaction is mediated by the host's immune system (specifically the protective effect of *H. polygyrus* towards *E. hungaryensis*), or *via* competition through shared host resources. Furthermore, we were previously not able to test for a reciprocal interaction of *E. hungaryensis* on *H. polygyrus* in the field due to difficulties in treating coccidia in the wild.

Here, we developed a wild-like laboratory system using laboratory-reared wood mice kept under standard laboratory conditions. This colony of wood mice was derived from wild-caught animals collected from woodlands near Liverpool, UK, which were taken into captivity and purposefully outbred for many generations. In addition, we used parasite isolates of both *H. polygyrus* and *E. hungaryensis* that were collected for a Scottish wood mouse population. Together with the laboratory-kept wood mice, using these wild-derived parasite isolates means that here, we were able to overcome many of the limitations of both laboratory and wild studies. First, we performed a controlled laboratory experiment to test (i) the effect of infection and coinfection on helminth and coccidian shedding and burdens, (ii) the effect of coinfection on protective immunity against *E. hungaryensis*, and (iii) antibody responses during single and coinfection. Next, we made use of two independent field experiments using the same host and parasite species as in the laboratory experiment to directly compare antibody levels between laboratory-kept and wild wood mice and to assess how wild conditions, including frequent exposure to a diverse parasite community, impact antibody levels. To measure the host immune response following parasite infection, we measured *H. polygyrus*-specific IgG1 and total fecal IgA levels. We chose to focus on these antibody types because they are both important during infection and can be measured in our non-model species using laboratory mouse reagents. IgG1 is the main antibody class responsible for worm clearance and establishment of protective immunity (Reynolds *et al*., 2012), whereas IgA is the main antibody class found at mucosal sites (Macpherson *et al*., 2012) and has been found to have anti-schizont and sporozoite activity during Eimeria infections in chickens (Davis *et al*., 1978; Trees *et al*., 1989).

To our knowledge, this study represents a unique and novel experimental approach to investigate coinfection dynamics using a combination of a mammal host and both micro- and macroparasites, where both host and parasites were either directly wild-derived or the recent descendants of wild-derived animals. We believe that this novel approach may help bridge the current gap between wild and laboratory studies in understanding the causes and consequences of coinfection.

## Methods

### Mice

We used a colony of *A. sylvaticus* that was derived from wild-caught animals collected from a woodland in the Wirral, UK around 5 years ago. Since then, the wood mice have been kept in standard laboratory conditions and purposely outbred to retain as much genetic variability among animals as possible. The wood mouse colony is currently housed at the University of Edinburgh under standard laboratory conditions. For this experiment, we used 12 females and 12 males aged between 8 and 23 weeks (average 15 weeks ± 0.9 s.e.). Prior to the start of the experiment, mice were housed in single-sex groups of 2–5 animals. After the start of the experiment, mice were housed individually in individual ventilated cages (Techniplast®, 1285L), with standard mouse chow and water *ad libitum*.

### Parasites

Transmission stages of the two parasite species used in this experiment (*H. polygyrus* L3 larvae and sporulated *Eimeria* spp. oocysts) were derived from feces collected from wild *A. sylvaticus*, trapped in September 2015 in a mixed woodland in Callendar park, Falkirk, UK (55.99°N, 3.77°W). To obtain *H. polygyrus* L3 larvae (the infective stage), we followed the protocol of Johnston *et al*. (2015) to hatch eggs from fecal material (Johnston *et al*., 2015). In short, fecal samples were soaked in water to soften and then mixed with water-soaked charcoal (DARCO®, 20–40 mesh particle size, granular, 242 268), which acted as a substitute for soil. A small amount of the feces-charcoal mix was spread thinly on a damp filter paper (Whatman No. 40 Filter Paper circles, 1440055) in a plastic petri dish. These fecal cultures were placed in a plastic container layered with damp filter paper to create a humid environment, and stored in the dark at 17 °C. After ~5 days, larvae migrated away from the charcoal mix to the edge of the filter paper and into the petri dish. Once larvae appeared in the petri dish, the filter paper was lifted onto a new petri dish and the larvae left behind were collected by flushing with tap water, and stored at 4 °C. Fecal cultures were checked every 48 h for the presence of new larvae.

To extract *Eimeria* spp. oocysts from feces, we modified the method used by Ryley *et al*. (2009). In short, fecal samples were soaked in water to soften and spun down at 4200 rpm for 10 min and the supernatant was removed. A 20–30 mL saturated salt solution was added to the fecal material, and samples were shaken vigorously to break up the pellets and spun down at 4200 rpm for 10 min. The supernatant containing the oocysts was collected in a fresh tube and the salt concentration in the tube was decreased by adding at least an equal amount of tap water to each tube. In order to pellet the oocysts, samples were spun again at 4200 rpm for 10 min. The pelleted oocysts were then washed three times with water and kept in a 2% potassium dichromate (K₂Cr₂O₇) solution to prevent bacterial and fungal growth and stored at 4 °C.

After transmission stages of both parasites were isolated from wild wood mice, the isolates were screened using PCR diagnostics to ensure that no other known mouse parasites or pathogens contaminated the field isolates (IDEXX Bioresearch, Germany). To ensure infectivity and to accumulate enough transmission stages, both *H. polygyrus* larvae and *Eimeria* spp. oocysts were passaged three times through colony-housed *A. sylvaticus*. In the case of *Eimeria* spp., this allowed us to selectively passage oocysts that morphologically matched oocysts of *E. hungaryensis*. We henceforth refer to the *Eimeria* spp. isolate used in the experiment as *E. hungaryensis*, however molecular confirmation of its identity is pending.

### Experimental design

**Fig. 1.**
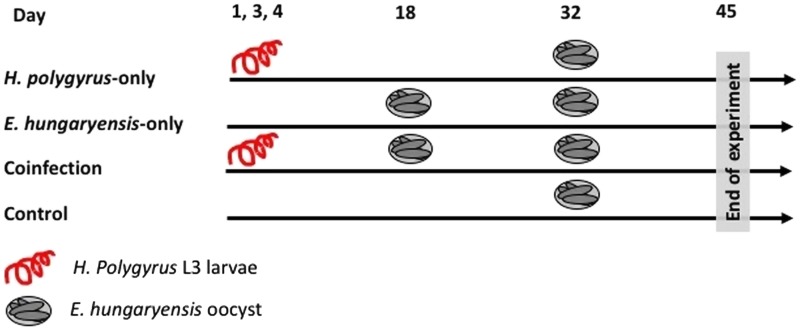
Schematic representation of infection schedules for each treatment group. Parasite dose per single inoculum were 80 L3 larvae in 200 *µ*L water for *H. polygyrus*, 500 sporulated oocysts in 200 *µ*L water for *E. hungaryensis*, or 200 *µ*L water without parasites.

On day 1 we randomly allocated mice to the following treatment groups: *H. polygyrus*-only, *E. hungaryensis*-only, coinfection and uninfected controls (Fig. 1). Animals in the *H. polygyrus*-only and coinfection groups received a dose of 80 *H. polygyrus* L3 larvae in 200 µl water *via* oral gavage on days 1, 3 and 4 (total 240 L3 larvae), while *E. hungaryensis*-only and control groups received an equivalent dose of water on the same days. We used three infection doses because we were limited in the number of wild *H. polygyrus* larvae available; the larval concentration in our isolates was too low to administer the inoculation in a single dose, and we decided against trying to concentrate the inoculum further due to the risk of losing any larvae during this process.

On day 18, *E. hungaryensis*-only and coinfection groups received a single dose of 500 sporulated *E. hungaryensis* oocysts in 200 *µ*L water *via* oral gavage, while *H. polygyrus*-only and control mice received an equivalent dose of water. On day 32, all groups were challenged with a dose of 500 sporulated *E. hungaryensis* oocysts (from the same starting inoculum) in 200 µl water *via* oral gavage. On day 45, all animals were culled using an overdose of CO_2_.

Over the course of the experiment, mice were sampled three times a week, starting on day 3. On each sampling occasion, we recorded body weight, took a small volume of host blood *via* tail bleed (2–10 µL) and collected a fresh fecal sample. The blood was spun at 12 000 rpm for 10 min and serum was stored at −20 °C. We collected 3–6 fecal pellets per mouse per sampling time point and these were dry frozen at −80 °C. The rest of the fecal pellets were weighed and stored in 10% buffered formalin at 4 °C to perform fecal egg counts by salt flotation (Dryden *et al*., 2005) and microscopy. After animals were euthanized on day 45, the number and sex of all adult *H. polygyrus* worms in the small intestine was counted for each mouse. The experiment was conducted in two blocks (replicates), with three animals randomly assigned to each treatment group per block, giving a total of six mice per treatment group (three males and three females).

One mouse failed to become infected with *E. hungaryensis* after the first *E. hungaryensis* challenge, but was successfully infected after the second *E. hungaryensis* challenge. We excluded this animal from our analysis on *E. hungaryensis* dynamics since we do not know the reason for the failed first infection. Additionally, animals in the second block were given their first *E. hungaryensis* challenge on day 16 instead of day 18 by mistake. This meant that oocyst shedding started on day 19 in the first block and on day 17 in the second block. However, this had no influence on peak oocyst shedding or total oocyst shedding for the first challenge, as there was no significant effect of block in any of the statistical models (Table 1).

**Table 1.** Modelling results for *H. polygyrus* worm burdens at day 45, last day of egg shedding, peak egg shedding and total egg shedding (*n* = 12). Each column represents a single model, each row represents a model covariate

Model covariate	Hp worms at day 45	Last Hp shedding	Peak Hp shedding	Total Hp shedding
Sex (male)	−0.14, *P* = 0.797	−0.05, *P* = 0.832	−0.07, *P* = 0.901	−1.35, *P* = 0.765
Age	0.02, ***P* =** 0.005**	0.002, *P* = 0.532	0.01, *P* = 0.926	0.09, *P* = 0.118
Mean body weight	0.02, *P* = 0.813	−0.01, *P* = 0.688	−0.11, *P* = 0.179	0.11, *P* = 0.843
Treatment (Hp)	−1.18, *P* < 0.0001***	−0.42, *P* = 0.001***	−0.10, *P* = 0.745	0.85, *P* = 0.728
Block (B)	0.45, *P* = 0.173	−0.12, *P* = 0.410	0.55, *P* = 0.173	5.24, *P* = 0.100

Each cell contains the covariate estimate and *P* value. Comparison levels for factors are given in brackets. *Hp* stands for *H. polygyrus.*

****P < 0.001, **P < 0.01, *P < 0.05, *P* < 0.1.

### Immunological methods

To measure *H. polygyrus*-specific IgG1 from serum samples, we coated plates (Nunc™ MicroWell™ 96-Well Microplates) with *H. polygyrus* excretory–secretory antigen [HES, supplied by R. M. Maizels, 1.0 *µ*g mL^−1^ (Johnston *et al*., 2015)] diluted in carbonate buffer overnight at 4 °C. Non-specific binding sites were blocked with Tris-buffered saline (TBS) containing 4% bovine serum albumin (BSA) at 37 °C for 2 h. Twofold serial dilution of serum samples were prepared in cluster tubes containing TBS-1% BSA, starting at 1:100. A serum sample of laboratory *M. musculus* that were artificially infected with *H. polygyrus* was added to each plate as a positive control (supplied by R. M. Maizels). After plates were washed with TBS-0.1% Tween 20, sample dilutions were added to the plates (50 *µ*L per well) and incubated overnight at 4 °C. After washing, 50 *µ*L goat anti-mouse IgG1-HRP detection antibody (Southern Biotech, Lot J6908-MC69), diluted 1:2000 in TBS-1%BSA was added to each well and incubated at 37 °C for 1 h in the dark. Plates were washed four times with TBS-Tween 20 and two times with _d_H2O, before 50 *µ*L tetramethylbenzidine (TMB) solution was added to each well. Plates were immediately covered to allow the enzymatic reaction to develop for 7 min and the reaction was stopped with 50 *µ*L 0.18 M sulphuric acid. Absorbance was measured at 450 nm. Cut-off values were calculated per plate as mean absorbance of blank wells plus three times the standard deviation of blank wells. The sample titre was determined as the denominator of the lowest sample dilution step that showed absorbance greater than the cut-off value.

For the fecal IgA ELISA, fecal extracts were prepared for each dry frozen fecal sample by soaking fecal pellets in a 3 to 1 volume of protease inhibitor solution (Complete Mini Protease Inhibitor Tablets, Roche, Cat No.: 11836153001). The extraction was then incubated for 1 h at room temperature, after which samples were centrifuged at 12 000 rpm for 5 min and the supernatant containing IgA removed. ELISA plates were coated with unlabelled goat anti-mouse IgA (Southern Biotech, Lot H7912-S233, 2 *µ*g mL^−1^) diluted in carbonate buffer overnight at 4 °C. Non-specific binding sites were blocked with TBS containing 4% BSA at 37 °C for 2 h. Fecal extracts were diluted 1:100 in cluster tubes containing TBS-1% BSA and added to the plates as triplicates, 50 *µ*L per well. Two 2-fold serial dilutions of standard antibody (Purified mouse IgA, *κ* isotype control, BD Pharmingen, Lot 3039828) at 50 *µ*L per well were added to each plate. Plates were incubated overnight at 4 °C and then after washing, 50 *µ*L goat anti-mouse IgA-HRP (Southern Biotech, UK, Lot G4512-V522D) diluted 1:4000 in TBS-1% BSA was added to each well and incubated at 37 °C for 1 h in the dark. Plates were washed four times with TBS-Tween and two times with _d_H2O, before 50 *µ*L TMB solution was added to each well and plates were immediately covered to allow the enzymatic reaction to develop for 7 min. The reaction was stopped with 50 *µ*L 0.18 M sulphuric acid and absorbance at 450 nm was measured. Sample concentrations of total fecal IgA were determined by fitting four-parameter logistic regression to standard curves using online software (www.elisaanalysis.com, ^©^Copyright 2012 Elisakit.com Pty Ltd.).

### Statistical analysis

All statistical analyses were performed using R software version 3.2.2 [R Core Team (2018), www.r-project.org].

### Laboratory experiment

To analyse *H. polygyrus* dynamics, we tested for differences between the *H. polygyrus*-only and coinfection group in (i) the number of worms recovered at the end of the experiment (continuous, worm counts), (ii) the duration of egg shedding (continuous, number of days), (iii) the peak egg shedding (continuous, eggs/gram feces rounded to the nearest integer) or (iv) total egg shedding (continuous, eggs/gram feces rounded to the nearest integer).

To analyse *E. hungaryensis* dynamics, we split the analysis in two parts: first, we measured oocyst shedding dynamics after the first challenge of the *E. hungaryensis*-only and coinfection group. Second, we measured oocyst shedding dynamics after the second *E. hungaryensis* challenge on day 32 (first challenge for the *H. polygyrus*-only and control groups), hence all the treatment groups were analysed. For both analysis parts, we asked whether there were differences between (i) the peak oocyst shedding and (ii) the total oocyst shedding (both variables are continuous, oocysts/gram feces rounded to the nearest integer).

To analyse *H. polygyrus*-specific IgG1 dynamics, we excluded the treatment groups that were not challenged with *H. polygyrus* before day 8 as no *H. polygyrus*-specific IgG1 antibodies were detected before this point in any of the treatments. We tested whether there were differences between (i) *H. polygyrus*-specific IgG1 levels (continuous, log-transformed IgG1 titres) throughout the experiment or (ii) *H. polygyrus*-specific IgG1 levels at the end of the experiment (day 45). We tested whether there were any differences between treatment groups in (i) total fecal IgA levels (continuous, ng mL^−1^) throughout the experiment across all the treatment groups, as all mice were assumed to be producing IgA because this non-specific antibody is known to be involved in gut homoeostasis (Macpherson *et al*., 2012).

In all the models described above we included the following covariates: host sex (factor, male or female), age at the start of the experiment (continuous, number of days), mean body weight over the experimental period (continuous, grams) and experimental block (factor, A or B). Depending on the response variable, we ran either linear models if the response variable was normally distributed, or generalized linear models (GLM) if the response variable was not. For the models that tested dynamic responses over time, we also included the covariate day (continuous, number of day in the experiment) and an interaction between treatment and day. Those models were run as linear mixed effect models, including animal ID as a random term to control for repeated measures on the same individual.

### Comparison laboratory vs *field*

In order to compare mean antibody levels between the lab and wild, we used data from three different sources. The first dataset came from the coinfection experiment described in this paper (henceforth called ‘laboratory’, *n* = 23), the second dataset came from a cross-sectional field experiment conducted in 2013 in the Wirral Peninsula, UK [henceforth called ‘Liverpool’, *n* = 54 (Clerc *et al*., 2018)] and the third dataset came from a longitudinal field experiment conducted in 2014 and 2015 in Falkirk, UK (henceforth called ‘Scotland’, *n* = 89, Clerc *et al*., in prep). For the laboratory dataset, we used IgA data collected between days 17 and 33, which corresponds to the time-point of the first *E. hungaryensis* challenge. The IgA data thereby should reflect more closely the IgA levels to be expected in the wild, where the probability of *H. polygyrus* and *E. hungaryensis* infection is high. Further, we used *H. polygyrus*-specific IgG1 data collected at the end of the experiment (day 45), which should more closely reflect what is to be expected in the wild (chronic helminth infection). For the Scotland dataset, we used data from first observations only, since the experiment subsequently involved anthelmintic treatment. Total fecal IgA and *H. polygyrus*-specific IgG1 levels were measured the same way for all datasets. In order to test the effect of *H. polygyrus* and *E. hungaryensis* infection on both antibody levels, we ran a linear model with either *H. polygyrus*-specific IgG1 or IgA as the response variable. The covariates included in each model were sex (factor, male or female), *H. polygyrus* infection (factor, yes or no), *E. hungaryensis* infection (factor, yes or no) and experiment (factor, lab, Liverpool or Scotland). To test whether the effect of *E. hungaryensis* on antibody levels depended on *H. polygyrus* infection, we also included an interaction between those two factors in each model.

## Results

### Laboratory experiment

**Fig. 2.**
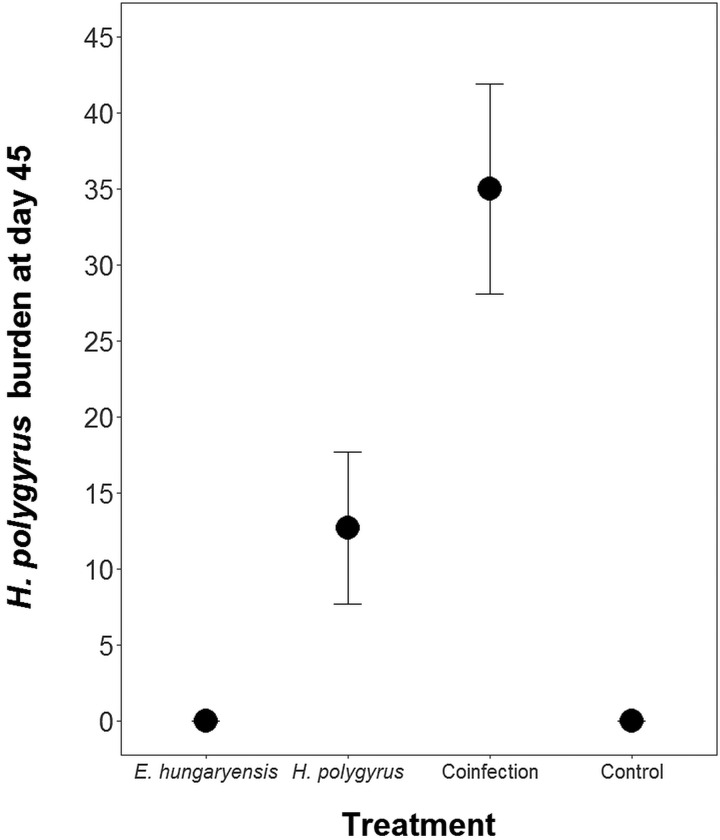
Number of adult *H. polygyrus* worms recovered from mice at day 45 of the experiment. Points represent means ± s.e.

**Fig. 3.**
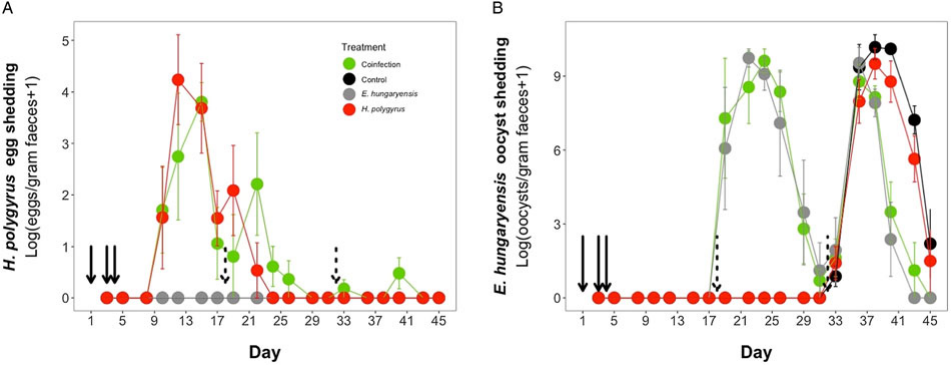
Parasite dynamics during the experimental period for (A) *H. polygyrus*, (B) *E. hungaryensis*. Points represent means ± s.e. Black, control; grey, *E. hungaryensis*-only; red, *H. polygyrus*-only; green, coinfection. Solid black arrows denote *H. polygyrus* challenge events, dashed black arrows denote *E. hungaryensis* challenge events.

Coinfected animals had on average over 2.5 times more worms than *H. polygyrus*-only infected animals by the end of the experiment (day 45; *H. polygyrus*-only: mean 13 worms ± 5 s.e.; coinfection: mean 35 worms ± 7 s.e.; negative binomial GLM: effect of *H. polygyrus*-only treatment: *E*_st_ = −1.18, *P* < 0.0001, Fig. 2, Table 1). We also found that age had a significant effect on the number of adult *H. polygyrus* worms harboured, with older mice harbouring fewer worms than younger mice. *H. polygyrus*-only and coinfected mice both started shedding *H. polygyrus* eggs on day 8, but the duration of egg shedding was significantly longer in coinfected animals than *H. polygyrus*-only (mean end of shedding on day 17 ± 1.5 s.e. *vs* 26 ± 4.5 s.e., respectively; Poisson GLM: effect of *H. polygyrus*-only treatment: *E*_st_ = −0.42, *P* = 0.001, Fig. 3A, Table 1). There was no significant difference in peak *H. polygyrus* shedding (*H. polygyrus*-only: mean 187 eggs/gram feces ± 47 s.e.; coinfection: mean 180 eggs/gram feces ± 44 s.e., Table 1) nor in total *H. polygyrus* shedding over the experimental period between *H. polygyrus*-only and coinfected mice (*H. polygyrus*-only: mean 336 eggs/gram feces ± 68 s.e.; coinfection: mean 260 eggs/gram feces ± 62 s.e., Table 1).

**Fig. 4.**
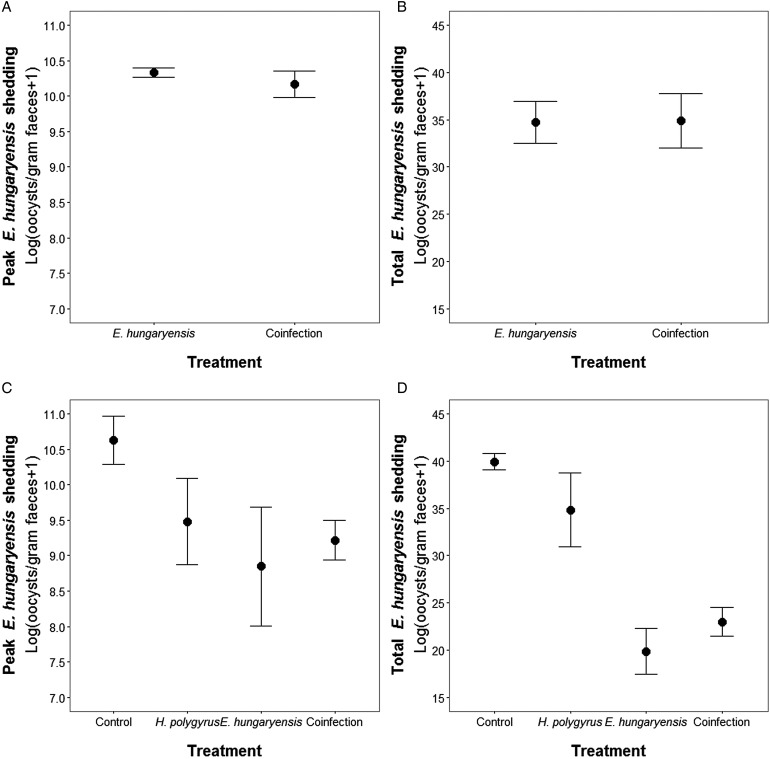
Peak and total *E. hungaryensis* shedding at different time points in the experiment. (A) Peak *E. hungaryensis* shedding during the first *E. hungaryensis* challenge, (B) total *E. hungaryensis* shedding during the first *E. hungaryensis* challenge, (C) peak *E. hungaryensis* shedding during the second *E. hungaryensis* challenge and (D) total *E. hungaryensis* shedding during the second *E. hungaryensis* challenge. Points represent means ± s.e.

During the first *E. hungaryensis* challenge (Fig. 3B day 18 onwards), there were no significant differences in peak oocyst shedding (Table 2, Fig. 4A) or total oocyst shedding (Table 2, Fig. 4B) between *E. hungaryensis*-only infected and coinfected mice. However, during the second challenge (Fig. 3B day 32 onwards), all treatment groups had at least 50% lower peak oocyst shedding than control mice (*E. hungaryensis*-only: 17 279 oocysts ± 8035 s.e.; coinfection: 12 110 oocysts ± 3087 s.e.; *H. polygyrus*-only: 27 505 oocysts ± 12 271 s.e.; control: 52 910 oocysts ± 14 349 s.e.; linear model: effect *of H. polygyrus*-only: *E*_st_ = −1.66, *P* = 0.006, effect of *E. hungaryensis*-only: *E*_st_ = −2.38, *P* = 0.001, effect of coinfection: *E*_st_ = −1.84, *P* = 0.003, Table 2, Fig. 4C). Further, total *E. hungaryensis* oocyst shedding was 63% lower during a second challenge compared with a first challenge (*E. hungaryensis*-only: 22 091 oocysts ± 8919 s.e.; coinfection: 17 160 oocysts ± 3342 s.e.; *H. polygyrus*-only: 58 733 oocysts ± 23 487 s.e.; control: 112 799 oocysts ± 34 366 s.e.; linear model: effect of *H. polygyrus*-only: *E*_st_ = −6.64, *P* = 0.056, effect of *E. hungaryensis*-only: *E*_st_ = −19.71, *P* < 0.0001, effect of coinfection: *E*_st_ = −17.76, *P* < 0.0001, Table 2, Fig. 4D).

**Table 2.** Modelling results for *E. hungaryensis* peak oocyst shedding for challenge 1 (*n* = 11), total oocyst shedding at challenge 1 (*n* = 11), peak oocyst shedding for challenge 2 (*n* = 23) and total oocyst shedding at challenge 2 (*n* = 23)

Model covariates	1^st^ peak Eh shedding	1^st^ total Eh shedding	2^nd^ peak Eh shedding	2^nd^ total Eh shedding
Sex (male)	0.30, *P* = 0.491	−5.85, *P* = 0.609	−0.36, *P* = 0.590	4.97, *P* = 0.241
Age	−0.01, *P* = 0.683	0.27, *P* = 0.644	−0.02, *P* = 0.009**	−0.10, *P* = 0.069^•^
Mean body weight	−0.10, *P* = 0.150	0.39, *P* = 0.802	0.09, *P* = 0.244	−0.16, *P* = 0.741
Treatment (Eh)	0.31, *P* = 0.265	−2.53, *P* = 0.718	−2.38, *P* = 0.001**	−19.71, *P* < 0.0001***
Treatment (Hp)	NA	NA	−1.66, *P* = 0.006**	−6.64, *P* = 0.056^•^
Treatment (Coinfection)	NA	NA	−1.84, *P* = 0.003**	−17.76, *P* < 0.0001***
Block (B)	−0.24, *P* = 0.762	10.99, *P* = 0.608	0.71, *P* = 0.133	−4.15, *P* = 0.154

Each column represents a single model, each row represents a model covariate. Each cell contains the covariate estimate and *P* value. Cells containing NA represent covariates that were not included in the model. Comparison levels for factors are given in brackets. *Hp* stands for *H. polygyrus*, *Eh* stands for *E. hungaryensis.*

****P < 0.001, **P < 0.01, *P < 0.05, ^•^*P* < 0.1.

**Fig. 5.**
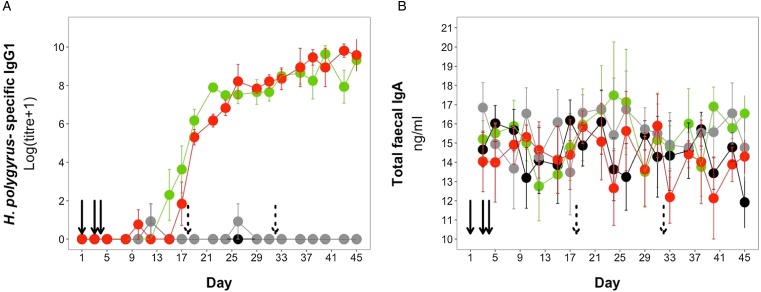
Antibody dynamics during the experimental period for (A) *H. polygyrus*-specific IgG1 and (B) total fecal IgA. Points represent means ± standard errors. Black, control; grey, *E. hungaryensis*-only; red, *H. polygyrus*-only; green, coinfection. Solid black arrows denote *H. polygyrus* challenge events, dashed black arrows denote *E. hungaryensis* challenge events.

*H. polygyrus*-specific IgG1 antibodies were detectable from day 12 onwards for mice in the coinfection group, and from day 15 onwards in the *H. polygyrus*-only group (Fig. 5A). After these time points, *H. polygyrus*-specific IgG1 titres increased steadily until around day 24, after which titres started to plateau (Fig. 5A). However, while the titre of *H. polygyrus*-specific IgG1 changed over time, there was no difference in the dynamics of *H. polygyrus*-specific IgG1, or in the final amount of *H. polygyrus*-specific IgG1 at the end of the experiment between the *H. polygyrus*-only and the coinfected mice (Fig. 5A, Table 3). Fecal IgA concentration varied substantially for all four treatment groups throughout the experimental period (Fig. 5B) and was not significantly different between treatment groups over time (Table 3).

**Table 3.** Analysis results for *H. polygyrus*-specific IgG1 dynamics, *H. polygyrus*-specific IgG1 levels at day 45 and total fecal IgA dynamics

Model covariates	Hp IgG1	Hp IgG1 day 45	Total IgA
Sex (male)	−0.69, *P* = 0.433	−0.31, *P* = 0.890	−0.68, *P* = 0.416
Age	−0.01, *P* = 0.733	0.01, *P* = 0.673	−0.004, *P* = 0.883
Mean body weight	0.06, *P* = 0.627	−0.16, *P* = 0.621	0.14, *P* = 0.141
Treatment (Hp)	−1.51, *P* = 0.161	−0.03, *P* = 0.982	−0.39, *P* = 0.693
Treatment (Eh)	NA	NA	−0.45, *P* = 0.681
Treatment (coinfection)	NA	NA	−0.62, *P* = 0.535
Day	0.26, *P* < 0.0001***	NA	−0.03, *P* = 0.244
Block (B)	0.58, *P* = 0.176	−1.67, *P* = 0.917	3.15, *P* < 0.0001***
Treatment (Hp) × day	0.04, *P* = 0.117	NA	0.007, *P* = 0.981
Treatment (Eh) × day	NA	NA	0.03, *P* = 0.426
Treatment (Coinf) × dDay	NA	NA	0.05, *P* = 0.121

Each column represents a single model, each row represents a model covariate. Each cell contains the covariate estimate and *P* value. Cells containing NA represent covariates that were not included in the model. Comparison levels for factors are given in brackets. *Hp* stands for *H. polygyrus*, *Eh* stands for *E. hungaryensis.*

****P < 0.001, **P < 0.01, *P < 0.05, *P* < 0.1.

### Comparison laboratory *vs* wild

**Fig. 6.**
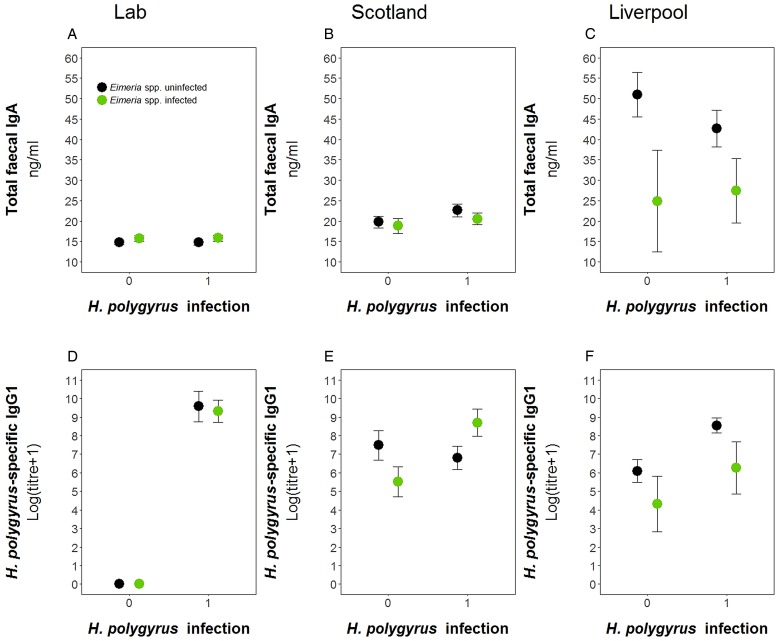
Comparison of antibody levels between laboratory and field studies. Top row, total fecal IgA; bottom row, *H. polygyrus*-specific IgG1. (A) and (D) represent the laboratory experiment, (B) and € represent the Scotland field study, and (D) and (F) represent the Liverpool field study. Data is split by *H. polygyrus* infection (*x*-axis) and *E. hungaryensis* (or *Eimeria* spp.) infection (green dots) or *E. hungaryensis* (or *Eimeria* spp.) uninfected (black dots). Points represent means ± s.e.

We used data from this laboratory experiment and two previous field experiments to test for the difference in antibody levels between the wild and laboratory in both single and coinfected hosts (Fig. 6). We found no difference in the prevalence of either parasite between the two field experiments for *H. polygyrus* (Scotland = 58.5%, Liverpool = 59.3%, *P* = 1.00) and *E. hungaryensis*/*Eimeria* spp. (Scotland = 32.0%, Liverpool = 18.5%, *P* = 0.101). However, Liverpool mice had higher IgA levels (linear model: effect of Liverpool origin: *E*_st_ = 25.81, *P* < 0.0001, Table 4, Fig. 6C) and Liverpool IgA levels were also more variable compared with the laboratory and Scotland samples. In the Scotland samples, IgA concentrations were highest in the autumn 2014 season (Spring 2014: 18.8 ± 1.4 ng mL^−1^, Autumn 2014: 24.1 ng mL ± 1.4 s.e., Spring 2015: 19.8 ng mL ± 1.0 s.e., ANOVA *F*_2,72_ = 3.65, *P* = 0.031). For *H. polygyrus*-specific IgG1, laboratory animals only had antibodies if they had been infected with *H. polygyrus* (Fig. 6D), whereas wild animals showed elevated *H. polygyrus*-specific IgG1 (Fig. 6E, F), even if they were not shedding *H. polygyrus* eggs at the time of sampling (linear model: effect of *H. polygyrus* infection: *E*_st_ = 1.52, *P* = 0.013, Table 4). Interestingly, *E. hungaryensis* had a negative effect on *H. polygyrus*-specific IgG1 levels in the field, but this effect was stronger in mice that were not currently shedding *H. polygyrus* eggs at the time of sampling (linear model: *H. polygyrus* × *E. hungaryensis*: *E*_st_ = 2.45, *P* = 0.045, Table 4).

**Table 4.** Analysis results for *H. polygyrus*-specific IgG1 and total fecal IgA comparison between laboratory, Liverpool and Scotland sites

Model covariates	Hp IgG1	Total IgA
Sex (male)	0.28, *P* = 0.585	−2.08, *P* = 0.384
Hp infection (yes)	1.52, *P* = 0.013*	−1.74, *P* = 0.544
Eh infection (yes)	−1.53, *P* = 0.064*	−7.42, *P* = 0.059^•^
Experiment (Scotland)	3.30, *P* < 0.0001***	5.21, *P* = 0.140
Experiment (Liverpool)	3.24, *P* < 0.0001***	25.81, *P* < 0.0001***
Hp infection × Eh infection	2.45, *P* = 0.045*	3.08, *P* = 0.547

Each column represents a single model, each row represents a model covariate. Each cell contains the covariate estimate and *P* value. Comparison levels for factors are given in brackets. *Hp* stands for *H. polygyrus*, *Eh* stands for *E. hungaryensis.*

****P < 0.001, **P < 0.01, *P < 0.05, ^•^*P* < 0.1.

## Discussion

By using the offspring of originally wild-derived wood mice and their naturally co-evolved parasites *H. polygyrus* and *E. hungaryensis*, we were able to demonstrate the impact of coinfection on the infection dynamics of both interacting parasites, as well as total and parasite-specific antibodies in a controlled environment. This enabled us to demonstrate, for the first time, that the interaction between these parasites in this system is reciprocal. Our results confirmed our previous wild experiment findings (Knowles *et al*., 2013), specifically that *H. polygyrus* has a negative effect on *E. hungaryensis* infection (but only on secondary challenge), but importantly were also able to demonstrate that *E. hungaryensis*, in turn, has a positive effect on *H. polygyrus* shedding and adult worm burdens. Further, thanks to our wild-like laboratory experiment, we directly compared antibody levels found in two independent field studies to those found under controlled laboratory conditions. This highlighted that wild mice had much higher baseline parasite-specific antibody levels, potentially suggesting much higher parasite exposure events, a series of trickle infections and/or a role for immune-memory in mediating parasite within-host interactions in the wild. Our results also highlight the crucial effect of coinfection for within-host parasite interactions, and enable us to gain a more mechanistic insight into the underlying causes governing this previously identified interaction, which was not possible from field-data alone due to unknown/uncontrolled levels of parasite exposure, nutritional status and infection histories.

One key finding of our study was that coinfection with *E. hungaryensis* increased the duration of *H. polygyrus*-egg shedding and led to 2.5 times higher adult worm burdens compared with singly infected mice. The higher worm burdens found in coinfected mice suggests that *E. hungaryensis* infection may reduce resistance towards *H. polygyrus.* Both singly and coinfected mice started shedding eggs at the same time and there was no difference in peak *H. polygyrus* egg shedding, suggesting that coinfection did not impact susceptibility and worm establishment. Instead we propose that coinfected mice were less able to expel adult worms from the gut, once coinfected, which resulted in much higher adult worm burdens and a longer period of egg shedding. A previous coinfection study using *H. polygyrus* and the bacterium *Bordetella bronchiseptica* conducted in laboratory mice found a similar effect; with no difference in worm burdens 12 days post-infection, but coinfected animals had significantly more worms at 24 and 48 days post-infection and a prolonged egg shedding period (Lass *et al*., 2013). We further found lower worm burdens in older compared with younger mice, which is consistent with recent findings from a wild study we conducted, and likely represents a less mature immune system in younger mice compared with older mice (Clerc *et al*., in review).

In our wild-like laboratory experiment, we also found that coinfection with *H. polygyrus* had no significant effect on *E. hungaryensis* oocyst shedding during primary infection (Fig. 2B green and grey lines). However, we then challenged all experimental groups, including the control and *H. polygyrus*-only groups, with *E. hungaryensis*, which showed that there was a negative effect of *H. polygyrus* infection during a secondary *E. hungaryensis* challenge leading to lower peak oocyst shedding in coinfected mice than in the control mice. Given that the negative effect of *H. polygyrus* on *E. hungaryensis* oocyst shedding was only found in the secondary challenge, this suggests that the interaction between the two parasites is not purely based on competition for shared host resources (Knowles *et al*., 2013), but likely also involves the host immune system. A study in laboratory mice found an effect of timing of worm coinfection on *Eimeria* infection (Rausch *et al*., 2010); mice were infected with *E. falciformis* either 6 or 28 days after *Heligmosomoides bakeri* infection and the results suggest that only early coinfection increased *E. falciformis* replication. This positive effect on *Eimeria* was accompanied by a reduced production of pro-inflammatory cytokines and an overall increase in the nematode-specific Th2 response, whereas chronic coinfection had no effect on *E. falciformis* replication (Rausch *et al*., 2010). A key difference between the Rausch *et al*. (2010) and our current study was that we used two parasites that physically overlap in their infection site (duodenum), whereas *H. bakeri* (duodenum) and *E. falciformis* (caecum) do not physically overlap. This suggests that in the case of coinfection of parasites that share the same niche, there is potential for more direct interaction between diverging immune cell populations as well as for tissue structural effects due to disruption of epithelial tissue integrity by the helminths (Boyett and Hsieh, 2014; Bramhall and Zaph, 2017). Specifically, in the case of *H. polygyrus*, tissue damage can occur when larvae are emerging from the gut epithelium and/or when feeding in the lumen as adults, which can limit the pool of suitable epithelial cells available for *E. hungaryensis* to infect. While, to our knowledge, no study has yet attempted to quantify the degree of available host cells for *Eimeria* infection in the case of helminth coinfection, a mathematical model of single-*Eimeria* infection found that host cell availability, specifically at high infection doses, could explain the so-called ‘crowding effect’, where *Eimeria* fecundity decreases with increased infection dose due to a smaller pool and lifespan of available epithelial cells (Johnston *et al*., 2001). It is possible that *H. polygyrus*-infection mimics the host cell availability of high-dose single *Eimeria* infections, due to tissue damage, but more work is needed to confirm this hypothesis. Ultimately, our results suggest that in order for *H. polygyrus* to exert a protective effect for the host against high *Eimeria* burden, the two parasites need to physically overlap during chronic *H. polygyrus* infection.

Overall, we show that the interaction between these two gut parasites is not uni-directional but reciprocal, with different outcomes for the epidemiology of each parasite: while *H. polygyrus* coinfection reduces the transmission potential of *E. hungaryensis via* a reduction in peak oocyst shedding (see also Knowles *et al*., 2013), *E. hungaryensis* coinfection can actually facilitate *H. polygyrus* transmission *via* increased worm survival and prolonged egg shedding. This result highlights that it is important to understand the direction in which parasites interact in order to make predictions about the causes and consequences of within-host interactions. Combining experiments conducted both in the laboratory and in the field represents a powerful tool to disentangle the underlying causes of parasite within-host interactions and their directions.

In addition to disentangling the within-host parasite interactions, we aimed to test whether mice were able to develop protective immunity to homologous *Eimeria* challenge, i.e. challenge with the same strain of *Eimeria* spp. In contrast to laboratory mice and chickens, where protective immunity towards homologous challenge is frequently observed (Smith *et al*., 2002; Steinfelder *et al*., 2005; Pogonka *et al*., 2010), wild mice are often found infected with one or multiple *Eimeria* species repeatedly over a long period of time, with prevalence usually ranging around 30–50% (Higgs and Nowell, 2000; Knowles *et al*., 2013). In our controlled laboratory infections of wood mice with *Eimeria*, although we found that peak and total *E. hungaryensis* oocyst shedding were lower in the secondary challenge, protective immunity was incomplete, as both the *E. hungaryensis*-only and coinfection group still shed significant *E. hungaryensis* oocysts after their second challenge. This lack of protective immunity towards reinfection may result from partial immunity to all strains (imperfect homologous immunity) or from complete immunity to only a subset of *E. hungaryensis* strains. Indeed, the *E. hungaryensis* isolate used in this experiment likely consisted of multiple genetically different *E. hungaryensis* strains and the original isolate was only passaged through colony housed wood mice three times. Heterologous immunity has been demonstrated in chickens, where hosts infected with one strain of *Eimeria maxima* were always protected against homologous challenge, whereas protection from heterologous challenge varied from 0 to 100% and further depended on host genotype (Smith *et al*., 2002). This highlights that natural levels of genetic diversity within an infective dose, which is highly likely to be the case in natural coccidian infection of wild wood mice (Higgs and Nowell, 2000), may be an important reason why hosts are unable to mount substantial protective immunity under natural conditions.

With regard to adaptive immunity to *H. polygyrus*, we found that *H. polygyrus*-specific IgG1 titres increased following helminth infection, but that coinfection had no effect on the magnitude of *H. polygyrus*-specific IgG1 in serum. This result resembles the findings of a laboratory mouse experiment that showed that coinfection with *Toxoplasma gondii* did not impact the production of *Fasciola hepatica*-specific IgG1 (Miller *et al*., 2009). In contrast, Fairlie-Clarke *et al*. (2010) showed reduced helminth-specific IgG1 levels in laboratory mice coinfected with either *Nippostrongylus brasiliensis* and *Plasmodium chabaudi*, or *Litomosoides sigmodontis* and *P. chabaudi* (Fairlie-Clarke *et al*., 2010). These conflicting results highlight the variable effect of microparasite coinfection on helminth-specific antibody production. *Heligmosomoides polygyrus*-specific IgG1 is important for reducing adult worm fecundity upon primary infection and plays a key role in worm expulsion after multiple *H. polygyrus*-challenges (McCoy *et al*., 2008). Because our study included only a single *H. polygyrus* challenge, the delay in *H. polygyrus* expulsion following *E. hungaryensis* coinfection may have been mediated by other immune factors. However, further work is needed to investigate the role of this antibody in secondary *H. polygyrus* challenge in our co-evolved system. We were surprised by the highly variable concentrations of total fecal IgA, which showed no discernible temporal pattern and no difference between treatment groups. This meant that we found a significant block effect in the IgA model, highlighting that the main portion of variation in IgA levels could not be explained by either the experimental treatment groups or any other important covariates. In coccidian infections, the precise role of IgA is still debated, but *E. maxima*-specific IgA levels have been shown to increase markedly at 8 days post infection in infected chickens (Yun *et al*., 2000). Additionally, parasite-specific IgA levels have been shown to rise after secondary *H. polygyrus*-infection, while total intestinal IgA levels stay constant after primary and secondary *H. polygyrus* infection (McCoy *et al*., 2008). In the future, measuring both total and parasite-specific fecal IgA will give more insight into how IgA may impact both coinfection and protective immunity to *Eimeria*.

By comparing the antibody levels observed in our controlled environment to those measured in two field experiments, we showed that *H. polygyrus*-infected laboratory mice produced more specific IgG1 compared with wild mice. Further, wild mice had elevated *H. polygyrus*-specific IgG1 levels, even when they were not currently shedding *H. polygyrus* eggs, whereas laboratory-kept mice produced no *H. polygyrus*-specific IgG1 in the absence of the parasite. This likely reflects the repeated infectious challenges that wild animals face in natural environments (Tinsley *et al*., 2012), and might suggest protective immunity in mice that were not shedding any *H. polygyrus* eggs at the time of sampling. We further observed that *Eimeria* infection had a significant negative effect on antibody levels in the wild, irrespective of the presence of *H. polygyrus* eggs (although the effect was stronger for *H. polygyrus*-specific IgG1 if *H. polygyrus* eggs were present). Interestingly, the negative effect of *Eimeria* infection on IgG1 titres was not consistent across field sites, as it was much stronger in the Liverpool population, where we also found overall higher IgA levels. This could, at least in part, be linked to seasonal effects; the Liverpool data were only collected in autumn, whereas the Scotland data were mainly collected in spring (two of the three trapping sessions), and IgA levels were significantly higher in the autumn session compared with the spring sessions in Scotland. In the spring, mice reach their peak reproductive period and a study in field voles has shown that this is the time point of lowest expression of pro- and anti-inflammatory immune markers, suggesting a trade-off between resource allocation to immunity and reproduction (Jackson *et al*., 2011). Further, strong seasonal changes in wood mice gut microbiome have been shown previously, with an overall more diverse bacterial community in late summer/autumn, and specifically higher abundances of *Alistipes* and *Heliobacter* (Maurice *et al*., 2015). Since commensal bacterial species are triggers of intestinal IgA expression (Macpherson *et al*., 2012), our finding of higher IgA levels in the autumn could suggest a higher capacity of the autumn-microbiome in triggering IgA expression, leading to higher baseline IgA levels irrespectively of parasite burdens. Alternatively, higher autumn IgA levels could also be caused by an accumulation of parasite exposure events during the spring and summer months, compared with mice sampled in the spring before a peak in parasite exposure. Both these hypotheses need further testing

By testing this natural host–parasite–parasite combination under controlled conditions, we offer a novel perspective on the within-host parasite interactions. Due to our unique experimental set-up (wild-derived hosts and co-evolved parasites that naturally interact), we were also able to compare our findings from the laboratory to previous findings from wild mouse populations, which confirmed our previous evidence of a negative interaction, but importantly identified a new positive interaction, demonstrating a previously unknown reciprocity in this interaction. Our results call for a more profound understanding of the effects of frequent exposure and force of infection on parasite and immune dynamics, which will enable the design of more realistic laboratory experiments and increase awareness of the importance of wild study systems.

## References

[ref1] Abolins SR, Pocock MJO, Hafalla JCR, Riley EM and Viney ME (2011) Measures of immune function of wild mice, *Mus musculus*. Molecular Ecology 20, 881–892. 21073587 10.1111/j.1365-294X.2010.04910.x

[ref2] Beura LK, Hamilton SE, Bi K, Schenkel JM, Odumade OA, Casey KA, Thompson EA, Fraser KA, Rosato PC, Filali-Mouhim A, Sekaly RP, Jenkins MK, Vezys V, Haining WN, Jameson SC and Masopust D (2016) Normalizing the environment recapitulates adult human immune traits in laboratory mice. Nature 532, 512–516. 27096360 10.1038/nature17655 PMC4871315

[ref3] Boyett D and Hsieh MH (2014) Wormholes in host defense: how helminths manipulate host tissues to survive and reproduce. PLoS Pathogens 10, e1004014. 24743351 10.1371/journal.ppat.1004014 PMC3990715

[ref4] Bramhall M and Zaph C (2017) Mastering gut permeability: new roles for old friends. European Journal of Immunology 47, 236–239. 28185248 10.1002/eji.201646842

[ref5] Clerc M, Devevey G, Fenton A and Pedersen AB (2018) Antibodies and coinfection drive variation in nematode burdens in wild mice. International Journal for Parasitology 48, 785–792. 29920254 10.1016/j.ijpara.2018.04.003

[ref6] Davis PJ, Parry SH and Porter P (1978) The role of secretory IgA in anti-coccidial immunity in the chicken. Immunology 34, 879–888. 350761 PMC1457199

[ref7] Dryden MW, Payne PA, Ridley R and Smith V (2005) Comparison of common fecal flotation techniques for the recovery of parasite eggs and oocysts. Veterinary Therapeutics 6, 15–28. 15906267

[ref8] Ezenwa VO and Jolles AE (2011) From host immunity to pathogen invasion: the effects of helminth coinfection on the dynamics of microparasites. Integrative and Comparative Biology 51, 540–551. 21727178 10.1093/icb/icr058

[ref9] Ezenwa VO, Etienne RS, Luikart G, Beja-Pereira A and Jolles AE (2010) Hidden consequences of living in a wormy world: nematode-induced immune suppression facilitates tuberculosis invasion in African Buffalo. American Naturalist 176, 613–624. 10.1086/656496 20849271

[ref10] Fairlie-Clarke KJ, Lamb TJ, Langhorne J, Graham AL and Allen JE (2010) Antibody isotype analysis of malaria-nematode co-infection: problems and solutions associated with cross-reactivity. BMC Immunology 11, 6. 20163714 10.1186/1471-2172-11-6 PMC2838755

[ref11] Friberg IM, Little S, Ralli C, Lowe A, Hall A, Jackson JA and Bradley JE (2013) Macroparasites at peripheral sites of infection are major and dynamic modifiers of systemic antimicrobial pattern recognition responses. Molecular Ecology 22, 2810–2826. 23379442 10.1111/mec.12212

[ref12] Gorsich EE, Ezenwa VO and Jolles AE (2014) Nematode-coccidia parasite co-infections in African Buffalo: epidemiology and associations with host condition and pregnancy. International Journal for Parasitology: Parasites and Wildlife 3, 124–134. 25161911 10.1016/j.ijppaw.2014.05.003 PMC4142258

[ref13] Graham AL (2008) Ecological rules governing helminth–microparasite coinfection. PNAS 105, 566–570. 18182496 10.1073/pnas.0707221105 PMC2206576

[ref14] Higgs S and Nowell F (2000) Population biology of Eimeria (Protozoa: Apicomplexa) in *Apodemus sylvaticus*: a capture’ recapture study. Parasitology 120, 355–363. 10811276 10.1017/s0031182099005545

[ref15] Jackson JA, Begon M, Birtles R, Paterson S, Friberg IM, Hall A, Lowe A, Ralli C, Turner A, Zawadzka M and Bradley JE (2011) The analysis of immunological profiles in wild animals: a case study on immunodynamics in the field vole, *Microtus agrestis*. Molecular Ecology 20, 893–909. 21059128 10.1111/j.1365-294X.2010.04907.x

[ref16] Johnston WT, Shirley MW, Smith AL and Granvenor MB (2001) Modelling host cell availability and the crowding effect in *Eimeria* infections. International Journal for Parasitology 31, 1070–1081. 11429170 10.1016/s0020-7519(01)00234-x

[ref17] Johnston CJ, Robertson E, Harcus Y, Grainger JR, Coakley G, Smyth DJ, McSorley HJ and Maizels R (2015) Cultivation of *Heligmosomoides polygyrus*: an immunomodulatory nematode parasite and its secreted products. Journal of Visualized Experiments (JoVE) 2015, e52412. 10.3791/52412 PMC4401400 25867600

[ref18] Knowles SC (2011) The effect of helminth co-infection on malaria in mice: a meta-analysis. International Journal for Parasitology 41, 1041–1051. 21777589 10.1016/j.ijpara.2011.05.009

[ref19] Knowles SC, Fenton A, Petchey OL, Jones TR, Barber R and Pedersen AB (2013) Stability of within-host-parasite communities in a wild mammal system. Proceedings Biological Sciences 280, 20130598. 23677343 10.1098/rspb.2013.0598 PMC3673050

[ref20] Lass S, Hudson PJ, Thakar J, Saric J, Harvill E, Albert R and Perkins SE (2013) Generating super-shedders: co-infection increases bacterial load and egg production of a gastrointestinal helminth. Journal of the Royal Society Interface 10, 20120588. 10.1098/rsif.2012.0588 PMC3565725 23256186

[ref21] Macpherson AJ, Geuking MB and McCoy KD (2012) Homeland security: IgA immunity at the frontiers of the body. Trends in Immunology 33, 160–167. 22410243 10.1016/j.it.2012.02.002

[ref22] Maizels RM and Gause WC (2014) How helminths go viral. Science 345, 517–518. 25082688 10.1126/science.1258443

[ref23] Maizels RM, Hewitson JP, Murray J, Harcus YM, Dayer B, Filbey KJ, Grainger JR, McSorley HJ, Reynolds LA and Smith KA (2012) Immune modulation and modulators in *Heligmosomoides polygyrus* infection. Experimental Parasitology 132, 76–89. 21875581 10.1016/j.exppara.2011.08.011 PMC6485391

[ref24] Maurice CF, Knowles SC, Ladau J, Pollard KS, Fenton A, Pedersen AB and Turnbaugh PJ (2015) Marked seasonal variation in the wild mouse gut microbiota. The ISME Journal 9, 2423–2434. 26023870 10.1038/ismej.2015.53 PMC4611506

[ref25] McCoy KD, Stoel M, Stettler R, Merky P, Fink K, Senn BM, Schaer C, Massacand J, Odermatt B, Oettgen HC, Zinkernagel RM, Bos NA, Hengartner H, Macpherson AJ and Harris NL (2008) Polyclonal and specific antibodies mediate protective immunity against enteric helminth infection. Cell Host & Microbe 4, 362–373. 18854240 10.1016/j.chom.2008.08.014

[ref26] Miller CM, Smith NC, Ikin RJ, Boulter NR, Dalton JP and Donnelly S (2009) Immunological interactions between 2 common pathogens, Th1-inducing protozoan *Toxoplasma gondii* and the Th2-inducing helminth *Fasciola hepatica*. PLoS ONE 4, e5692. 19478853 10.1371/journal.pone.0005692 PMC2682559

[ref27] Moreno PG, Eberhardt MA, Lamattina D, Previtali MA and Beldomenico PM (2013) Intra-phylum and inter-phyla associations among gastrointestinal parasites in two wild mammal species. Parasitology Research 112, 3295–3304. 23820605 10.1007/s00436-013-3509-x

[ref28] Pedersen AB and Antonovics J (2013) Anthelmintic treatment alters the parasite community in a wild mouse host. Biology Letters 9, 20130205. 23658004 10.1098/rsbl.2013.0205 PMC3730629

[ref29] Pedersen AB and Fenton A (2007) Emphasizing the ecology in parasite community ecology. Trends in Ecology & Evolution 22, 133–139. 17137676 10.1016/j.tree.2006.11.005

[ref30] Pogonka T, Schelzke K, Stange J, Papadakis K, Steinfelder S, Liesenfeld O and Lucius R (2010) CD8 + cells protect mice against reinfection with the intestinal parasite *Eimeria falciformis*. Microbes and Infection 12, 218–226. 20034589 10.1016/j.micinf.2009.12.005

[ref46] R Core Team (2018) R: A language and environment for statistical computing. Vienna, Austria: R Foundation for Statistical Computing https://www.R-project.org/

[ref31] Rausch S, Held J, Stange J, Lendner M, Hepworth MR, Klotz C, Lucius R, Pogonka T and Hartmann S (2010) A matter of timing: early, not chronic phase intestinal nematode infection restrains control of a concurrent enteric protozoan infection. European Journal of Immunology 40, 2804–2815. 20809519 10.1002/eji.201040306

[ref32] Reese TA, Bi K, Kambal A, Filali-Mouhim A, Beura LK, Burger MC, Pulendran B, Sekaly RP, Jameson SC, Masopust D, Haining WN and Virgin HW (2016) Sequential infection with common pathogens promotes human-like immune gene expression and altered vaccine response. Cell Host & Microbe 19, 713–719. 27107939 10.1016/j.chom.2016.04.003 PMC4896745

[ref33] Reynolds LA, Filbey KJ and Maizels RM (2012) Immunity to the model intestinal helminth parasite *Heligmosomoides polygyrus*. Seminars in Immunopathology 34, 829–846. 23053394 10.1007/s00281-012-0347-3 PMC3496515

[ref34] Rosshart SP, Vassallo BG, Angeletti D, Hutchinson DS, Morgan AP, Takeda K, Hickman HD, McCulloch JA, Badger JH, Ajami NJ, Trinchieri G, de Villena FPM, Yewdell JW and Rehermann B (2017) Wild mouse gut microbiota promotes host fitness and improves disease resistance. Cell 171, 1015-+. 29056339 10.1016/j.cell.2017.09.016 PMC6887100

[ref35] Ryley JF, Meade R, Hazelhurst J and Robinson TE (2009) Methods in coccidiosis research: separation of oocysts from faeces. Parasitology 73, 311–326. 10.1017/s0031182000046990 13340

[ref36] Salgame P, Yap GS and Gause WC (2013) Effect of helminth-induced immunity on infections with microbial pathogens. Nature Immunology 14, 1118–1126. 24145791 10.1038/ni.2736 PMC4955540

[ref37] Seabloom EW, Borer ET, Gross K, Kendig AE, Lacroix C, Mitchell CE, Mordecai EA and Power AG (2015) The community ecology of pathogens: coinfection, coexistence and community composition. Ecology Letters 18, 401–415. 25728488 10.1111/ele.12418

[ref38] Smith AL, Hesketh P, Archer A and Shirley MW (2002) Antigenic diversity in *Eimeria maxima* and the influence of host genetics and immunization schedule on cross-protective immunity. Infection and Immunity 70, 2472–2479. 11953384 10.1128/IAI.70.5.2472-2479.2002 PMC127903

[ref39] Steinfelder S, Lucius R, Greif G and Pogonka T (2005) Treatment of mice with the anticoccidial drug Toltrazuril does not interfere with the development of a specific cellular intestinal immune response to *Eimeria falciformis*. Parasitology Research 97, 458–465. 16163562 10.1007/s00436-005-1464-x

[ref40] Tate AT and Graham AL (2015) Dynamic patterns of parasitism and immunity across host development influence optimal strategies of resource allocation. The American Naturalist 186, 495–512. 10.1086/682705 26655573

[ref41] Telfer S, Birtles R, Bennett M, Lambin X, Paterson S and Begon M (2008) Parasite interactions in natural populations: insights from longitudinal data. Parasitology 135, 767–781. 18474121 10.1017/S0031182008000395 PMC2952918

[ref42] Tinsley R, Stott L, York J, Everard A, Chapple S, Jackson J, Viney M and Tinsley MC (2012) Acquired immunity protects against helminth infection in a natural host population: long-term field and laboratory evidence. International Journal for Parasitology 42, 931–938. 22906507 10.1016/j.ijpara.2012.07.006

[ref43] Trees AJ, Karim MJ, Mckellar SB and Carter SD (1989) *Eimeria tenella*: local antibodies and interactions with the sporozoite surface. Journal of Protozoology 36, 326–333. 2671342 10.1111/j.1550-7408.1989.tb05521.x

[ref44] Turner AK, Begon M, Jackson JA, Bradley JE and Paterson S (2011) Genetic diversity in cytokines associated with immune variation and resistance to multiple pathogens in a natural rodent population. PLoS Genetics 7, e1002343. 22039363 10.1371/journal.pgen.1002343 PMC3197692

[ref45] Yun CH, Lillehoj HS and Lillehoj EP (2000) Intestinal immune responses to coccidiosis. Developmental and Comparative Immunology 24, 303–324. 10717295 10.1016/s0145-305x(99)00080-4

