# Parasitic nematodes simultaneously suppress and benefit from coccidian coinfection in their natural mouse host – CORRIGENDUM

**DOI:** 10.1017/S0031182019000623

**Published:** 2019-06-06

**Authors:** Melanie Clerc, Andy Fenton, Simon A. Babayan, Amy B. Pedersen

The authors apologise for the following error:

It was mistakenly stated that the colony of *A. sylvaticus* used in the study was “derived from wild-caught animals collected from a woodland in the Wirral, UK around 5 years ago”.

Rather, the outbred colony was originally established by J. Clarke in 1995 at the Department of Zoology, University of Oxford, and was maintained with occasional introductions of wild wood mice. The wood mouse colony moved to the University of Liverpool, Faculty of Veterinary Science around 10 years ago (Hughes *et al.*, 2010), and then a subset of the wood mice was brought to the University of Edinburgh from Liverpool around 6 years ago.

**Hughes DJ, Kipar A, Sample JT and Stewart JP** (2010) Pathogenesis of a Model Gammaherpesvirus in a natural host. *Journal of Virology* **84**(8), 3949–3961.

In addition, an update is available for a reference that was in preparation at the time of publication (given in the text as ‘Clerc et al. in prep’ and ‘Clerc et al. in review’):

**Clerc M, Babayan SA, Fenton A and Pedersen AB** (2019) Age affects antibody levels and anthelmintic treatment efficacy in a wild rodent. *International Journal for Parasitology: Parasites and Wildlife* **8**, 240–247. DOI: 10.1016/j.ijppaw.2019.03.004
